# Morphological Differences between Circulating Tumor Cells from Prostate Cancer Patients and Cultured Prostate Cancer Cells

**DOI:** 10.1371/journal.pone.0085264

**Published:** 2014-01-08

**Authors:** Sunyoung Park, Richard R. Ang, Simon P. Duffy, Jenny Bazov, Kim N. Chi, Peter C. Black, Hongshen Ma

**Affiliations:** 1 Department of Mechanical Engineering, University of British Columbia, Vancouver, British Columbia, Canada; 2 Department of Biology, Kwantlen Polytechnic University, Surrey, British Columbia, Canada; 3 Vancouver Prostate Centre, Vancouver General Hospital, Vancouver, British Columbia, Canada; 4 BC Cancer Agency, Vancouver Cancer Centre, Vancouver, British Columbia, Canada; 5 Department of Urologic Science, University of British Columbia, Vancouver, British Columbia, Canada; University of Illinois at Chicago, United States of America

## Abstract

Circulating tumor cell (CTC) enumeration promises to be an important predictor of clinical outcome for a range of cancers. Established CTC enumeration methods primarily rely on affinity capture of cell surface antigens, and have been criticized for underestimation of CTC numbers due to antigenic bias. Emerging CTC capture strategies typically distinguish these cells based on their assumed biomechanical characteristics, which are often validated using cultured cancer cells. In this study, we developed a software tool to investigate the morphological properties of CTCs from patients with castrate resistant prostate cancer and cultured prostate cancer cells in order to establish whether the latter is an appropriate model for the former. We isolated both CTCs and cultured cancer cells from whole blood using the CellSearch® system and examined various cytomorphological characteristics. In contrast with cultured cancer cells, CTCs enriched by CellSearch® system were found to have significantly smaller size, larger nuclear-cytoplasmic ratio, and more elongated shape. These CTCs were also found to exhibit significantly more variability than cultured cancer cells in nuclear-cytoplasmic ratio and shape profile.

## Introduction

Circulating tumor cells (CTCs) have been implicated as potential seeds of cancer metastasis and are therefore of great importance in research, disease management, and drug development [1]–[3]. Established methods for capturing these cells, such as the Veridex CellSearch® system (Raritan, NJ, USA), rely on affinity capture of the epithelial cell surface antigen, EpCAM, followed by fluorescence labeling of intracellular cytokeratin (CK) [4]–[6]. While CTC identification and enumeration, based on epithelial biomarker expression, can be used to predict poor clinical outcome [7]–[10] this strategy may be prone to underestimation of CTC number because of epithelial-to-mesenchymal transition [11]–[14], poor expression of these factors in some tumor types [14], or changes in expression of these factors following chemotherapy [15]. These limitations may be particularly relevant, given that the appearance of mesenchymal CTCs is associated with disease progression [16] and the inclusion of additional criteria CTC identification may be a valuable supplement to conventional CellSearch® CTC enumeration.

In addition to their expression of tumor antigens, it is broadly accepted that CTCs have distinct biomechanical characteristics, including larger size than leukocytes, greater nuclear to cytoplasmic (N:C) ratio, as well as distinct nuclear morphology [17]. Numerous strategies have been developed to enrich for CTCs based on these characteristics [18]. CTCs have been isolated using density gradient centrifugation [19] or by size, using micropore filtration [20]–[22]. Recently, microfluidic technologies have achieved superior CTC capture efficiency and enrichment using approaches such as hydrodynamic chromatography [23]–[28], microfluidic filtration [29]–[31], and dielectrophoresis [32]–[35]. The development of these technologies typically used cultured cancer cells as a morphological model for clinical CTCs. However, while cancer cells and some CTCs have common biophysical features [17], CTCs may exhibit distinct morphological characteristics, depending on the type of originating tumor [36]. An alternative strategy would be to incorporate biomechanical characterization with the more established antigen-based CellSearch® CTC enumeration strategy.

We developed a software tool to analyze the cytomorphological properties of cancer cells. We employed this tool to examine both patient CTC and model cancer cell line morphology, following CellSearch® enrichment. These results will provide important data to aid in CTC identification based on combined antigen and biomechanical criteria [36] as well as in choosing appropriate models for optimization of biomechanical CTC enrichment.

## Materials and Methods

### Blood Sample Collection

Blood samples from healthy donors and patients with metastatic castrate resistant prostate cancer (CRPC) were obtained with written informed consent and collected using protocols approved by the UBC Clinical Ethics Review board (http://research.ubc.ca/ethics/clinical-research-ethics-board). The CRPC patients included in this study ranged in age, from 53–83 years, and PSA levels, from 21.1-2200 μg/L (Table S1). Blood samples in both cases are collected and stored in CellSave® Vacutainer tubes (Becton Dickinson, Raritan, NJ).

### Isolation and Enumeration of CTCs by CellSearch

CTCs isolation and enumeration were performed using the CellSearch® system as previously described [4], [5], [37]. Briefly, blood samples were drawn into 10 ml CellSave Vacutainer tubes (Becton Dickinson) containing proprietary anticoagulant and preservative. Samples were maintained at room temperature and processed within 48 hours after collection. The CellSearch® system captures EpCAM expressing cells using antibody-coated magnetic beads and then labels these cells with fluorescent dyes, such as DAPI, CD45, and cytokeratins, in order to distinguish potential CTCs from leukocytes. After immunomagnetic capture and fluorescence staining, images of candidate CTCs are obtained in brightfield and three fluorescence channels (DAPI, CD45, and cytokeratins). The captured images are segmented into multiple smaller images each containing a single cell and reassembled in a panel in software. Finally, a certified technician positively identifies the CTCs by reviewing the size, shape, and fluorescence intensity of each candidate cell.

### Cell Culture and Processing

Human prostate cancer cell lines including LNCaP (ATCC: CRL-1740), DU145 (ATCC: HTB-81), and C4-2 (ATCC: CRL-1595) were propagated in culture using RPMI-1640 medium (HyClone, Logan, UT) with 10% fetal bovine serum at 37°C with 5% CO_2_. PC3 (ATCC: CRL-1435) cells was cultured similarly, but using DMEM (HyClone, Logan, UT) medium instead. Cultured cancer cells were spiked into 7.5 ml of blood from a healthy donor into CellSave® Vacutainer tubes and processed within 48 hours identically as the patient specimen.

### Image Processing

**Figure 1:**
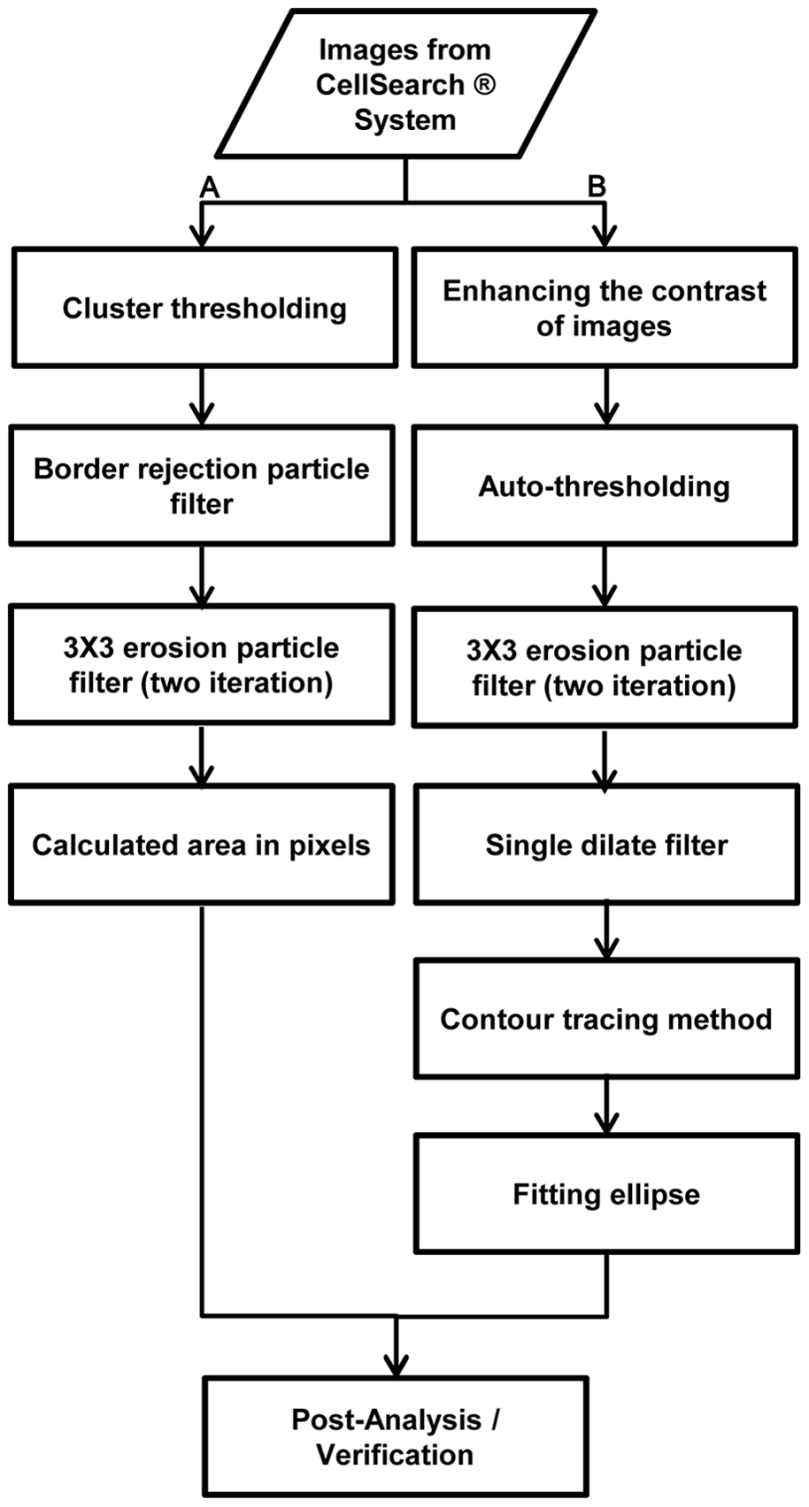
Image data processing using LabView software. Labview software performs the series of operations on large varying mages datasets provided by the CellSearch® system. Two parallel filtering and measurements, such as calculating area in pixels (A) and estimating the best-fit ellipse (B) are performed for optimal performance and results.

To study the morphology of CTCs and cultured cancer cells, we exported images of individual cells from the CellSearch® system and analyzed them using a software program we developed using LabView (Figure S1; National Instruments, Austin, TX). The images were square matrices with sizes ranging from 80 to 200 pixels and formatted as portable network graphics (PNG) files as 8 bit mono or 24 bit color composites (Figure S1). To calculated area in pixels, the images were initially processed using cluster thresholding to detect bright objects to match the auto-exposure performed by the CellSearch® system (Figure 1A). Particles with pixels in contact with the edge of the image frame were removed using a border rejection particle filter to eliminate cells incompletely bounded by the images (Figure S1). Multi-particle images were also eliminated using watershed segmentation. Debris particles were removed using two-iterations of a 3×3 erosion particle filter. Cell and nuclear size was determined by counting the number of above-threshold pixels in the cytokeratin and DAPI channel respectively. Results for the cell and nuclear size calculation were filtered to remove cells with improbable nuclear sizes, which we define as the nuclear area exceeding 95% of the cell area. These processing steps rejected 209 out of 732 images, or 28.5% of the total. The majority of the rejected images contained cell fragments or poor quality images (Figure S2).

### Eccentricity of Cell Shape Measurement

To analyse the eccentricity of cell shape, an ellipse was fitted to the outline of the cell. The overview of estimating the best-fit ellipse using LabView software is shown in Figure 1B. To enhance detection of the cell outline, the images were contrast-enhanced before auto-thresholding. Three-iterations of a 3×3 erosion filter, as well as a single dilate filter were performed to smooth the edges of the particles. Fitting was performed on a binary image using the contour tracing method to search for the longest ellipse perimeter within the image (Figure S1). After fitting the ellipse the results were visually confirmed by the operator. To quantify eccentricity of the cell shape, we calculated the elongation factor (EF), defined as the ratio between the major and minor axes of the best-fit ellipse.

### Size Measurement Calibration

To calibrate size measurements from the CellSearch® images, we separately measured the size of the cultured prostate cancer cells in suspension using the CEDEX XS imaged-based cell analyzer (Roche, Germany). Grown cultured cancer cells are trypsinized and re-suspended in the culture medium. Cell counts were evaluated using a 1∶1 dilution of cell suspension in trypan blue (Gibco, Grand Island, NY). A 10 μl of cell suspension is loaded on the Smart Slide (Roche, Germany), and then read to measure the cell diameter. The conversion factor from pixels to micrometers can be determined using the following equation,
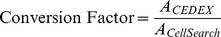



The size of CTCs from patient samples was estimated by products of the conversion factor and area of CTCs measured from CellSearch® images.

### Nuclear Cytoplasmic Ratio Measurement

The nuclear cytoplasmic ratio is defined as the ratio of nuclear area (A_N_) to cytoplasmic (A_C_) area, where A_C_ is considered as the area of the cell excluding A_N_.
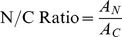



### Sample Selection

CTCs analyzed in this study were obtained from baseline blood samples of consenting patients diagnosed with metastatic castration resistant prostate cancer that were chemotherapy-naïve and enrolled onto a randomized phase II clinical trial of a novel agent [38]. We collected all of the images showing DAPI+CK+CD45- events that were identified by CellSearch® for the 83 subjects enrolled in the trial. Based on the likelihood that a small fraction of these events represented legitimate CTCs we restricted our analysis to 19 patients who had >40 DAPI+CK+CD45- events. Three of these patients were further excluded because of low quality of images. CTC enumeration was independently determined for the remaining 16 patients by a CellSearch®-qualified technician and the counts ranged from 11 to 106 CTCs/7.5 ml, with a median value of 41.5 CTCs/7.5 ml. After excluding unsuitable images (Figure S2), because they could not be interpreted by our software, a total of 523 CTCs from prostate cancer patients were analyzed along with 800 cultured cancer cells from the four prostate cancer cell lines.

## Results and Discussion

### Cell Size

**Figure 2:**
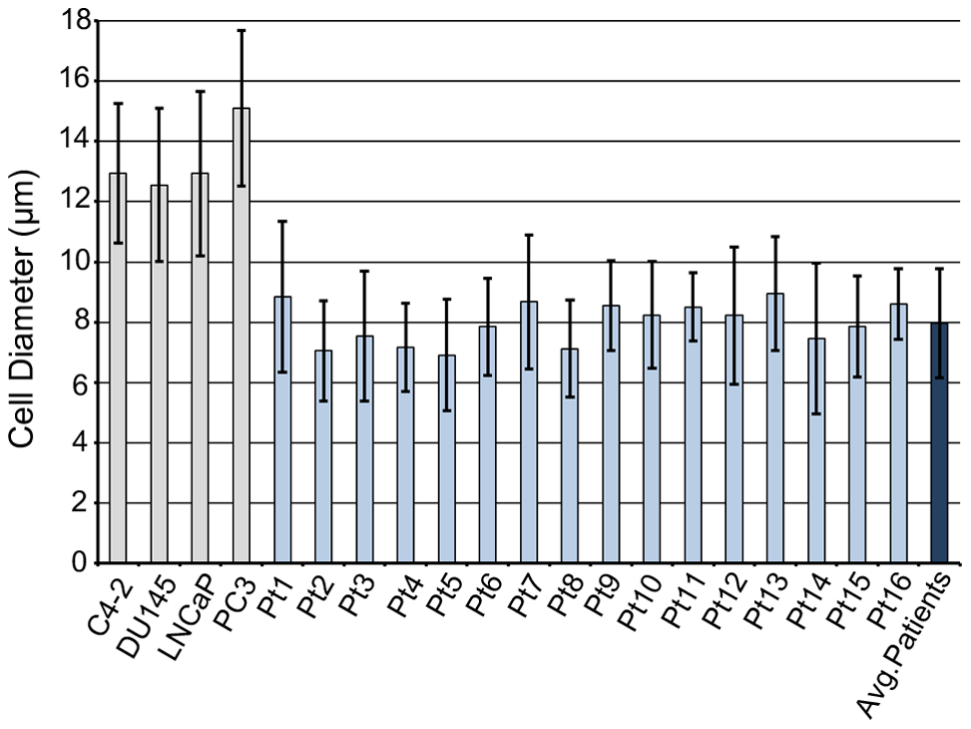
Diameters of CTCs from prostate cancer patients (pre-treatment) and cultured prostate cancer cells. The average diameter of CTCs (7.97 µm) was significantly smaller than cultured cancer cells (13.38 µm) (p<0.001).

Analyzing images of cells processed using the CellSearch® system and calibrated against standard microscopy, we found significant size differences between prostate cancer CTCs and cultured prostate cancer cells (Figure 2). Specifically, the average diameter of CTCs captured by CellSearch®, among our 16 patients, is just over half that of the cultured cancer cells, with 7.97±1.81 µm for CTCs and 13.38±2.54 µm for cultured cancer cells (p<0.001) respectively. While Coumans and colleagues [21] employed a micropore filtration strategy for the enrichment of both cancer cell lines and patient-derived CTCs, they characterized the biomechanical properties of the same EpCAM^+^CK^+^CD45^−^ CTC population presented in this study. Their analysis reported that prostate CTCs were smaller than those of breast or colorectal cancers, however, they estimated that prostate cancer CTCs were ∼25% larger than our current report. While this discrepancy may represent cell stress imposed in sample processing by immunocapture, in the current study, and micropore filtration, in the former, this difference may also have arisen from the different strategies employed to measure cell size. Coumans used a Coulter pipette for size calibration, which was less precise than image analysis for small length scale (<10 µm) size estimates. Furthermore, our estimate of cell diameter is consistent with the small mean cell volume reported by Ligthart and colleagues [36], as well as another recent study showing LNCaP total cell area is 1.6-fold greater than that of EpCAM^+^CK^+^CD45^−^ prostate cancer CTCs [39]. It is also interesting to note that the optimal pore size used in previous studies for filtration based capture of CTCs was 8 µm, which coincides with our estimated cell diameter of 7.97 µm [21], [22], [29], [40]. Micropore filtration strategies have reported as high as 90% CTC recovery [40] but have relatively poor sample purity [41].The similarity in size between CRPC CTCs examined in this study and leukocytes, this may represent a fundamental limitation of filtration-based strategies. This limitation can be potentially overcome by enrichment strategies that combine CTC enrichment based on a combination of cell size and deformability [29]–[31].

We also considered the possibility of our patient selection criteria (CTC count >40) may have biased for a greater number of smaller CTCs. While the selected patients were chemotherapy-negative, they would have participated in a range of therapeutic interventions and would represent patients in the late stages of the disease. Due to these or other unique physiological burdens within our patient cohort, caution should be exercised in generalizing these results to all CRPC CTCs. However, we observed no correlation between CTC cell size and cell count (Table S1 and Figure S3) that would otherwise suggest that disease severity affects cell size. Interestingly, while other studies have reported a high degree of heterogeneity in CTC cell size [21], [36], [42], [43], our size estimation based on microscopic analysis demonstrated that the inter-patient variation of the mean cell size was quite small, ranging from 7.05 µm to 8.94 µm with a median of 8.04 µm. Furthermore, the currently accepted criterion used by the CellSearch® system to validate CTCs is that their size must be larger than neighbouring leukocytes [17]. However, this size definition was largely determined based on CTCs derived from breast cancer [44]–[47] that have a median cell diameter of 13.1 µm [21]. Our observation that CTCs from patients with CRPC are significantly smaller in size (∼8 µm) suggests that these conventional criteria for CTC identification may underestimate the true CTC count.

Similarly, our observation that prostate cancer EpCAM^+^ CTCs are consistently smaller than cultured cancer cells is potentially important for emerging label-free CTC enrichment strategies. Firstly, enrichment of CTCs based on size alone may have limited efficacy for the capture of the smaller CTCs found in CRPC patients because they will not be as clearly discriminable from patient leukocytes. While larger cancer cells are typically used to demonstrate the efficacy of these techniques, such as HELA (>20 µm), LNCaP (18 µm), MCF-7 (>15 µm), MDA-231 (15 µm) [21], [48], [49], cultured cancer cells, such as L1210 mouse lymphoma cells (10 µm), with smaller diameter may represent better models for CTC enrichment [31], [50], [51]. Secondly, the contribution of the nucleoplasm to cell stiffness is 10-fold greater than the cytoplasm [52]; CTC enrichment strategies that capture CTCs based on size and deformability may prove to be superior to those that sort based on size alone.

### Cell Shape

**Figure 3:**
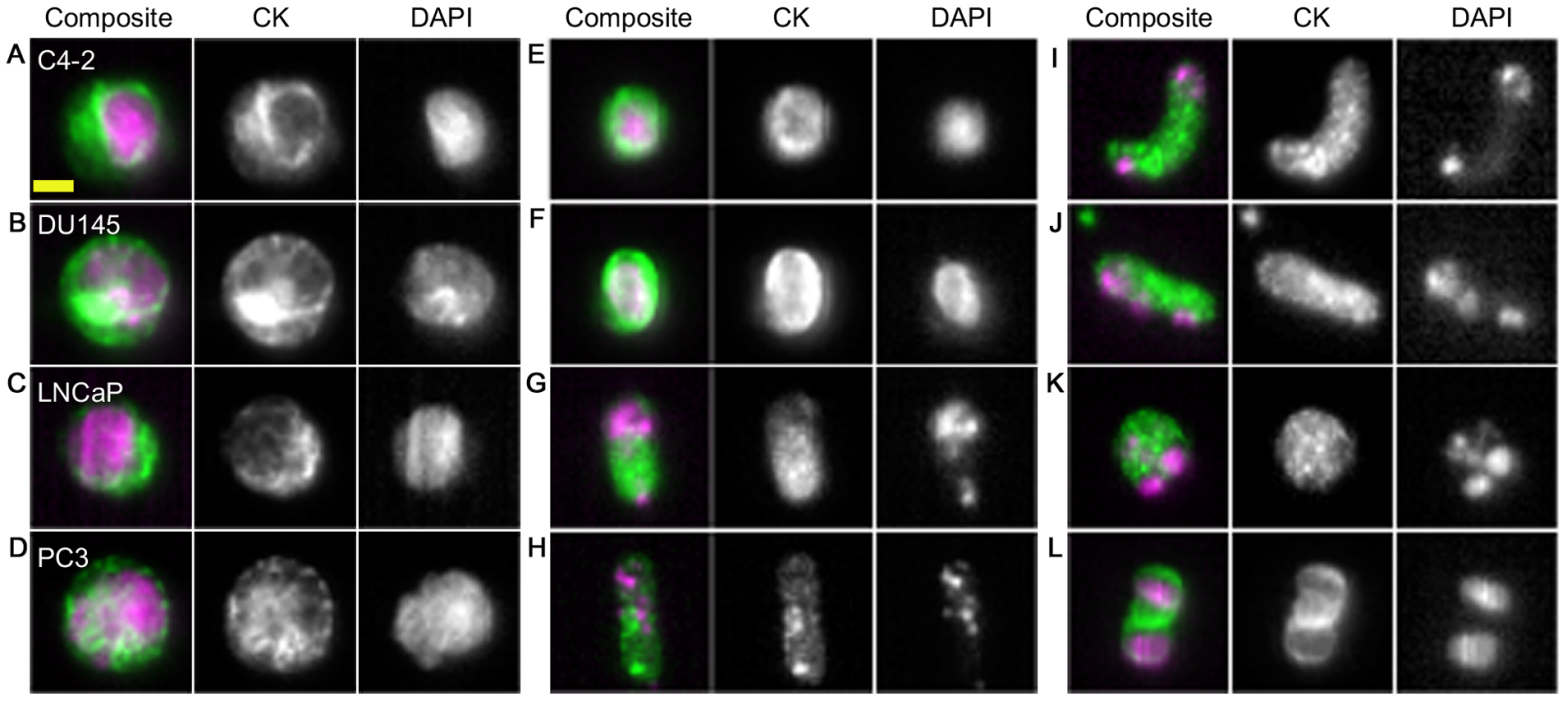
Example images of cultured prostate cancer cell (A–D) and CTCs from prostate cancer patients (E–L) captured using the CellSearch® system. CTCs were noticeably smaller than cultured cancer cells (A–D). Cultured cancer cells were mostly round with regular cell and nuclear shapes. The nucleus was typically centered and surrounded by cytokeratin (E–L). CTCs exhibited highly variable shapes, including round (E), oval (F), elongated (G–J), and clusters (L). Non-round and multi-nucleate cells were sometimes observed (G–K). The yellow scale bar is 5 µm in length.

**Figure 4:**
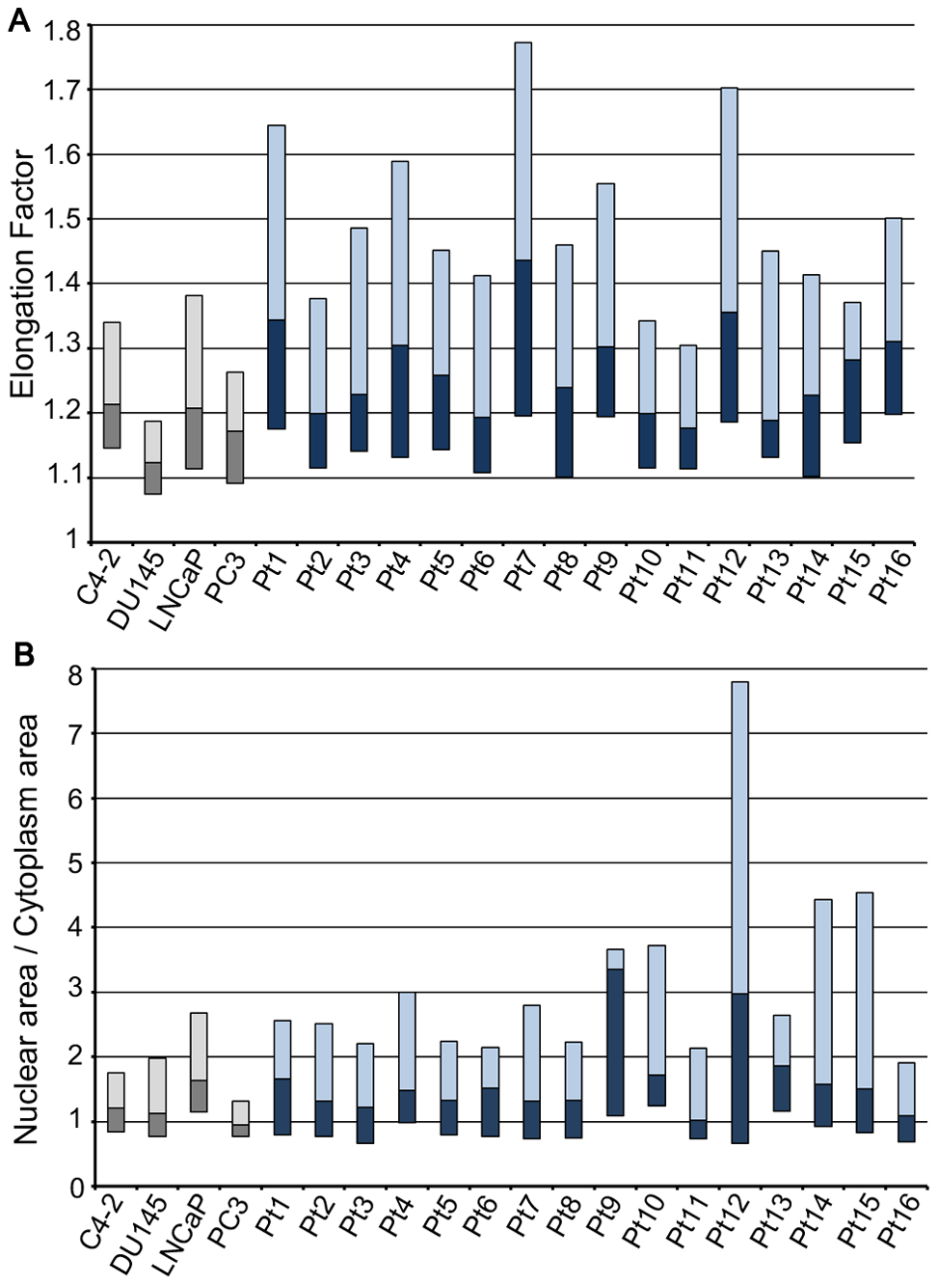
Elongation Factor and Nuclear Cytoplasmic Ratios. A: Elongation factor (EF) of CTCs from prostate cancer patients compared with cultured prostate cancer cells. The median EF of CTCs was generally greater with significant inter- and intra-patient variability. B: Nuclear cytoplasmic (N/C) ratios of CTCs from prostate cancer patients compared with cultured prostate cancer cells. Median with upper and lower quartiles is shown for each sample. The median N/C ratio for CTCs was generally greater with significant inter- and intra-patient variability.

The use of cultured cancer cells spiked into blood from healthy donors may model the separation of these cells from hematological cells that differ in cell size but the common pre-treatment of these cells with trypsin, to dissociate them from tissue culture flasks, or sample processing using the CellSearch affinity capture strategy may also dramatically influence the cell shape. Through comparison of trypsinized cultured cells and CRPC CTCs, following CellSearch® CTC enrichment, we evaluated whether these cultured cells are appropriate models for patient-derived CTCs. In contrast to cultured cells, that were generally round in shape, CTCs exhibited significant shape variability with many cells having a more elongated shaped (Figure 3). We quantified the eccentricity of the cell shape using the elongation factor (EF), defined as the ratio between the major and minor axes of the best-fit ellipse. As shown in Figure 4A, CTCs were significantly more elongated than cultured cancer cells with an average median EF of 1.27 compared 1.17 for cultured cancer cells (p<0.05). This observation is consistent with other studies that report significant pleomorhpism among CTCs [21], [36], [42], [43]. One possible cause for the diversity in cell morphology are apoptotic events associated with CTC dissemination [53]. However, the cytomorphological changes observed in CTCs may represent functional changes associated with interactions between CTC and endothelium or cellular elongation associated with vascular transport [54], [55]. Interestingly, cytomorphological abnormality of CTCs has been correlated to poor clinical outcome in metastatic breast, colorectal, and prostate cancer. [36].

### Nuclear Cytoplasmic Ratio

The nuclear cytoplasmic ratio (N/C) is defined as the ratio of the apparent nuclear area and the apparent cell area with the nucleus subtracted. Compared to cultured cancer cells, CTCs are expected to have larger N/C because of their smaller cell size and likely larger nuclear size due to possible chromosomal abnormalities. We found the median N/C for all cultured cancer cells to be 1.12 while average median N/C ratio from the CTC of patients, enriched by CellSearch® system to be 1.43. This observation further underscores the potential efficacy of deformability-based CTC enrichment, as the nucleoplasm contributes 10-fold more to cell stiffness than does the cytoplasm [52]. Furthermore, as shown in Figure 4B, CTCs showed significantly greater N/C variability than cultured cancer cells. Considering that the N/C ratio of CTCs correlates to poor disease outcome [36], it may be speculated that, within this heterogeneous population, there are cell subpopulations with greater metastatic potential. If so, then perhaps a more relevant measure of disease status is the count of a certain subpopulation of CTCs rather than the count of all CTCs as used currently [9], [56].

### Cell Shrinkage

**Figure 5:**
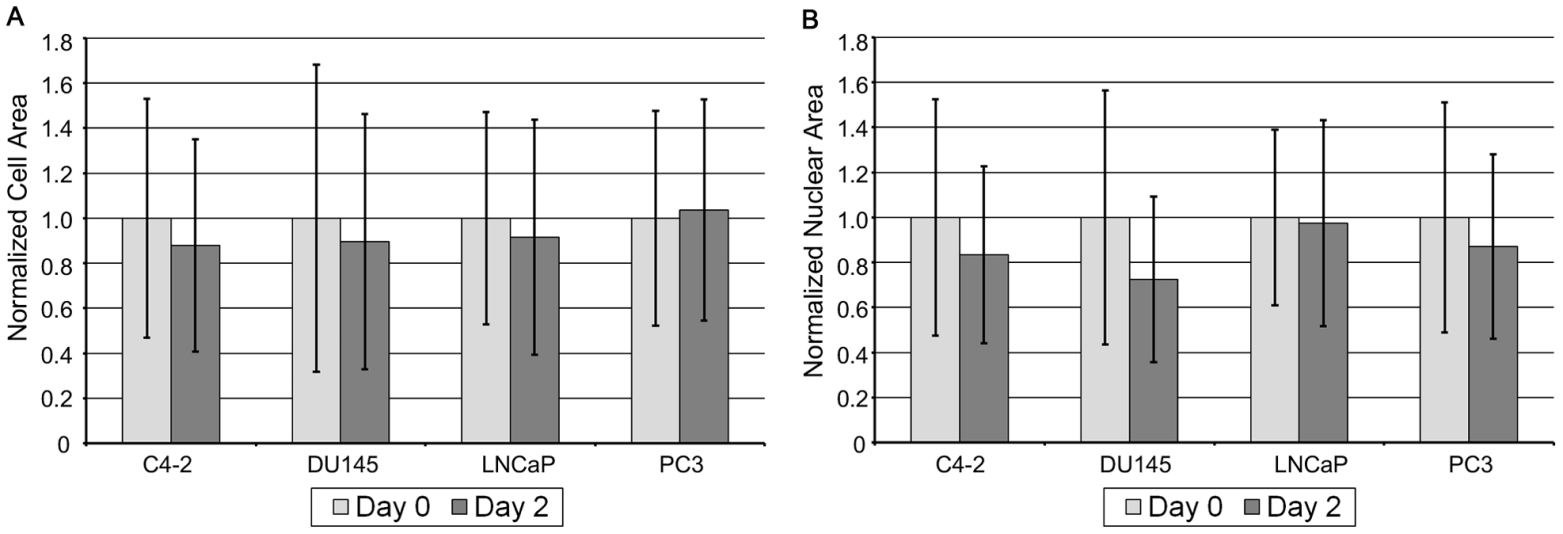
Changes in cell and nucleus after two days of storage in the CellSave tubes. A: The diameter of cultured prostate cancer cells decreased ∼6% on average. B: The nuclear diameter of cultured prostate cancer cells decreased ∼10% on average.

One concern associated with measuring cell size using the CellSearch® system is whether storing cell samples in the CellSave® tubes modifies the size of the cell and nucleus. To investigate, we compared cultured cancer cells spiked into blood from healthy donors processed immediately with the same cells processed after 48 hours of storage. The cell diameter was found to decrease by ∼6%, while the nuclear diameter was found to decrease by ∼10% from 0 to 48 hours (Figure 5). This result gives an estimate of the variability of the measured cell morphology parameters resulting from sample storage time, but cannot explain the significant differences in the morphology of CTCs and cultured cancer cells, or the variability found within each CTC sample.

## Conclusion

In conclusion, CTCs isolated from castrate resistant prostate cancer patients, using the CellSearch® system, were smaller in size, more elongated in shape, and had greater N/C when compared to cultured cancer cells. CTCs also showed significantly greater variability in shape and N/C. While the system only captures EpCAM-high cells, CTC images from CellSearch® enumeration are widely available and this analytical strategy could be applied to identify characteristic morphological features of CellSearch®-enriched CTCs. The morphological differences between cultured cell lines and CTC need to be considered in the design and testing of devices that isolate CTC in a label-free fashion based on cytomorphological criteria.

## Supporting Information

Figure S1 Screen-shot of the LabView® program developed to analyze images obtained from the CellSearch® system. The program acquires the images for each CTC candidate. A selected image (highlighted in yellow) is analyzed to measure the area in pixels. An ellipse is fitted to this image and overlaid on top of the original image for checking. Parameters for intermediate image processing steps, as well as statistics for the whole collection are also displayed.
(DOCX)

Figure S2 Rejected images of cell fragments from CTC identification. These images of cell fragments commonly appeared during image analysis and were not included in the CTC count or cell size measurements. Typical CTC fragments include a nucleus partly covered by cytokeratin, or a nucleus completely separated from cytokeratin. These fragments likely originated from CTCs undergoing apoptosis.
(DOCX)

Figure S3 Cell size versus CTC count. There appeared to be no correlation between CTC cell size and cell count for CTCs identified by CellSearch from patients with metastatic castrate resistant prostate cancer. The cell size ranged from 6.9 µm to 8.95 µm; while the CTC count varied from 11 to 106.
(DOCX)

Table S1 **Patient information summary.** All patients were diagnosed with metastatic castration resistant prostate cancer (mCRPC). There was no significant correlation between PSA level and size of CTCs.
(DOCX)
