# Supplementary figures and images for: Morphological Differences between Circulating Tumor Cells from Prostate Cancer Patients and Cultured Prostate Cancer Cells
Source: PLoS One. 2014 Jan 8;9(1):e85264. doi: 10.1371/journal.pone.0085264 (PMC3885705; doi:10.1371/journal.pone.0085264)

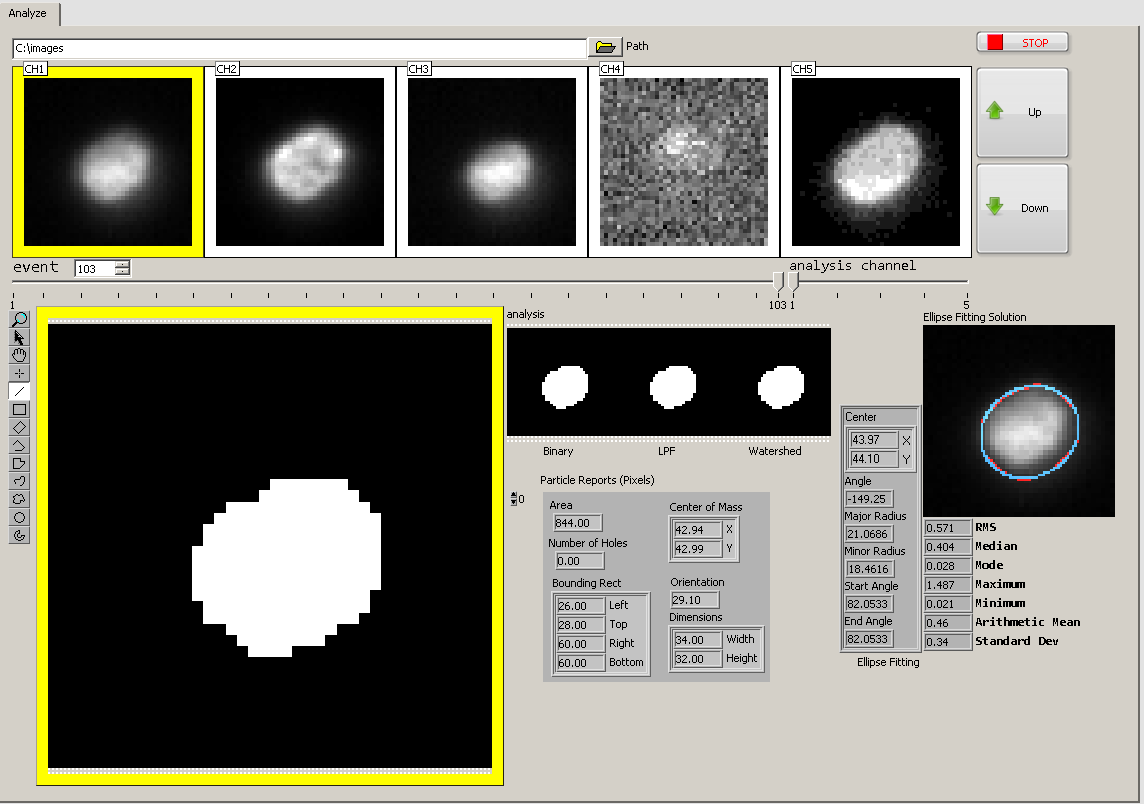

Supplement: Figure S1 — Screen-shot of the LabView® program developed to analyze images obtained from the CellSearch® system. The program acquires the images for each CTC candidate. A selected image (highlighted in yellow) is analyzed to measure the area in pixels. An ellipse is fitted to this image and overlaid on top of the original image for checking. Parameters for intermediate image processing steps, as well as statistics for the whole collection are also displayed. (DOCX) [file pone.0085264.s001.docx]

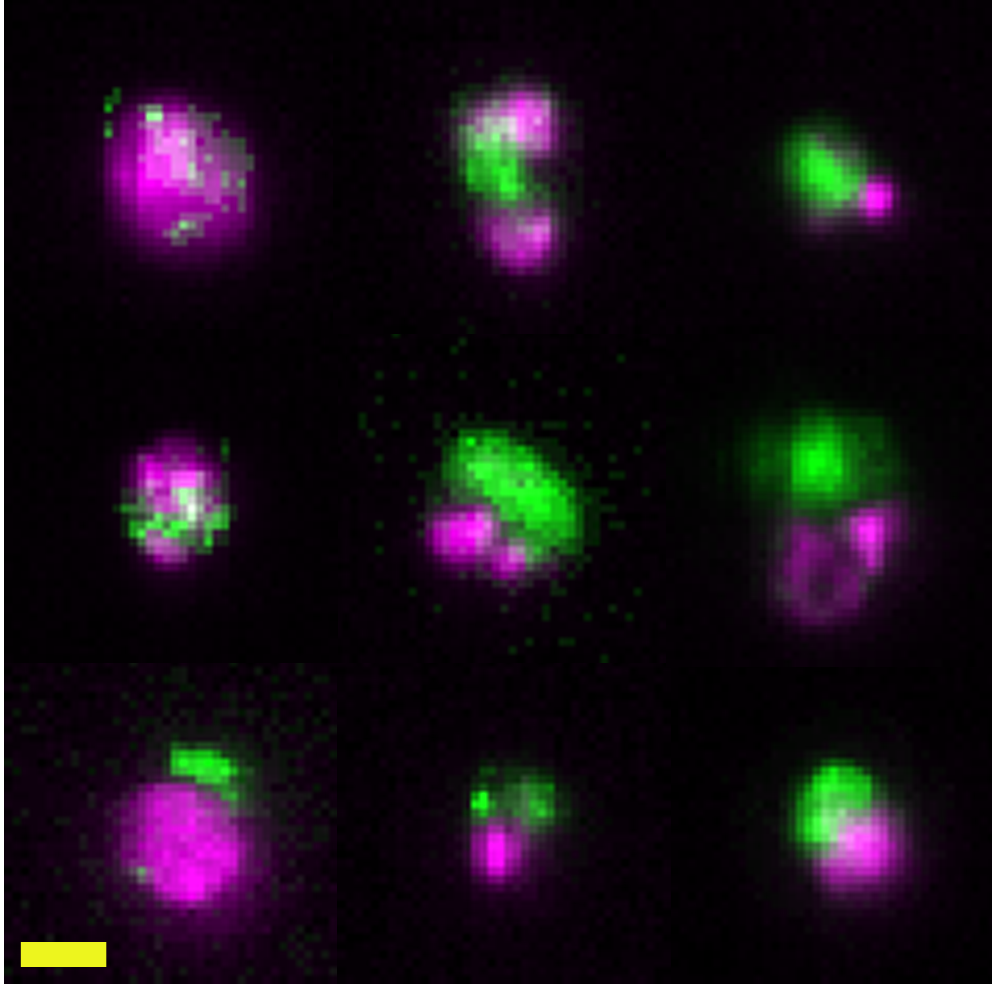

Supplement: Figure S2 — Rejected images of cell fragments from CTC identification. These images of cell fragments commonly appeared during image analysis and were not included in the CTC count or cell size measurements. Typical CTC fragments include a nucleus partly covered by cytokeratin, or a nucleus completely separated from cytokeratin. These fragments likely originated from CTCs undergoing apoptosis. (DOCX) [file pone.0085264.s002.docx]

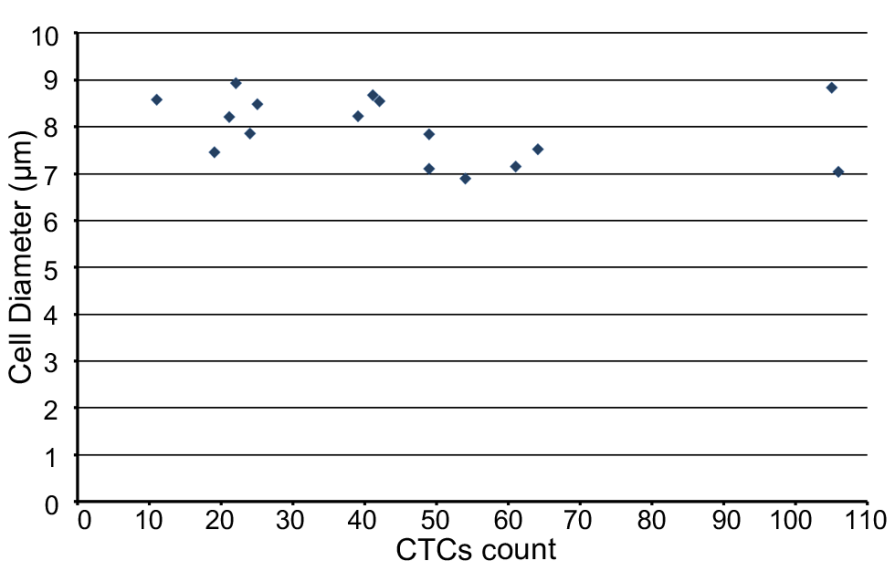

Supplement: Figure S3 — Cell size versus CTC count. There appeared to be no correlation between CTC cell size and cell count for CTCs identified by CellSearch from patients with metastatic castrate resistant prostate cancer. The cell size ranged from 6.9 µm to 8.95 µm; while the CTC count varied from 11 to 106. (DOCX) [file pone.0085264.s003.docx]
