# Supplementary material for: Morphological Differences between Circulating Tumor Cells from Prostate Cancer Patients and Cultured Prostate Cancer Cells
Source: PLoS One. 2014 Jan 8;9(1):e85264. doi: 10.1371/journal.pone.0085264 (PMC3885705; doi:10.1371/journal.pone.0085264)
Supplement: Table S1 — Patient information summary. All patients were diagnosed with metastatic castration resistant prostate cancer (mCRPC). There was no significant correlation between PSA level and size of CTCs. (DOCX) [file pone.0085264.s004.docx]

| Patient ID | Age | PSA level (μg/L) | mCRPC | Chemotherapy naive | Cell size in diameter (μm) | SD | CTCs (no. per 7.5ml) |
| --- | --- | --- | --- | --- | --- | --- | --- |
| 1 | 69 | 28 | Y | Y | 8.84 | 2.50 | 105 |
| 2 | 77 | 156.4 | Y | Y | 7.05 | 1.67 | 106 |
| 3 | 62 | 21.1 | Y | Y | 7.54 | 2.15 | 64 |
| 4 | 67 | 456.9 | Y | Y | 7.16 | 1.47 | 61 |
| 5 | 77 | 184.7 | Y | Y | 6.90 | 1.85 | 54 |
| 6 | 62 | 21.85 | Y | Y | 7.85 | 1.62 | 49 |
| 7 | 68 | 160 | Y | Y | 8.67 | 2.23 | 41 |
| 8 | 72 | 53 | Y | Y | 7.11 | 1.61 | 49 |
| 9 | 71 | 2200 | Y | Y | 8.55 | 1.49 | 42 |
| 10 | 67 | 50 | Y | Y | 8.24 | 1.77 | 39 |
| 11 | 83 | 17.6 | Y | Y | 8.50 | 1.13 | 25 |
| 12 | 53 | 146 | Y | Y | 8.22 | 2.28 | 21 |
| 13 | 66 | 240 | Y | Y | 8.95 | 1.89 | 22 |
| 14 | 75 | 637.8 | Y | Y | 7.46 | 2.50 | 19 |
| 15 | N/A | N/A | Y | Y | 7.86 | 1.68 | 24 |
| 16 | N/A | N/A | Y | Y | 8.60 | 1.17 | 11 |
